# Supplementary material for: Pyruvate Dehydrogenase Kinase 1 inhibition mediated oxidative phosphorylation enhancement in cartilage promotes osteoarthritis progression
Source: BMC Musculoskelet Disord. 2023 Jul 20;24:597. doi: 10.1186/s12891-023-06585-6 (PMC10357736; doi:10.1186/s12891-023-06585-6)
Supplement: Supplementary file 1 — Additional file 1. [file 12891_2023_6585_MOESM1_ESM.zip › MF.Gsea.1653627316242/GOMF_UBIQUITIN_LIGASE_SUBSTRATE_ADAPTOR_ACTIVITY.html]

Details for gene set GOMF\_UBIQUITIN\_LIGASE\_SUBSTRATE\_ADAPTOR\_ACTIVITY[GSEA]

|  || Dataset | 55457\_collapsed\_to\_symbols.cls.cls #OA\_versus\_normal.cls.cls #OA\_versus\_normal\_repos |
| Phenotype | cls.cls#OA\_versus\_normal\_repos |
| Upregulated in class | OA |
| GeneSet | GOMF\_UBIQUITIN\_LIGASE\_SUBSTRATE\_ADAPTOR\_ACTIVITY |
| Enrichment Score (ES) | 0.39976338 |
| Normalized Enrichment Score (NES) | 1.284668 |
| Nominal p-value | 0.16475973 |
| FDR q-value | 0.40881878 |
| FWER p-Value | 1.0 |
Table: GSEA Results Summary

  

Fig 1: Enrichment plot: GOMF\_UBIQUITIN\_LIGASE\_SUBSTRATE\_ADAPTOR\_ACTIVITY      
 Profile of the Running ES Score & Positions of GeneSet Members on the Rank Ordered List

  

| SYMBOL | TITLE | RANK IN GENE LIST | RANK METRIC SCORE | RUNNING ES | CORE ENRICHMENT || 1 | DET1 | DET1 partner of COP1 E3 ubiquitin ligase [Source:HGNC Symbol;Acc:HGNC:25477] | 50 | 0.989 | 0.1407 | Yes |
| 2 | PDCD6 | programmed cell death 6 [Source:HGNC Symbol;Acc:HGNC:8765] | 87 | 0.933 | 0.2743 | Yes |
| 3 | KLHDC2 | kelch domain containing 2 [Source:HGNC Symbol;Acc:HGNC:20231] | 126 | 0.879 | 0.3998 | Yes |
| 4 | PEF1 | penta-EF-hand domain containing 1 [Source:HGNC Symbol;Acc:HGNC:30009] | 1784 | 0.387 | 0.3277 | No |
| 5 | FBXO7 | F-box protein 7 [Source:HGNC Symbol;Acc:HGNC:13586] | 2327 | 0.323 | 0.3328 | No |
| 6 | PRAME | PRAME nuclear receptor transcriptional regulator [Source:HGNC Symbol;Acc:HGNC:9336] | 3235 | 0.227 | 0.2956 | No |
| 7 | VHL | von Hippel-Lindau tumor suppressor [Source:HGNC Symbol;Acc:HGNC:12687] | 4142 | 0.143 | 0.2461 | No |
| 8 | KLHDC3 | kelch domain containing 3 [Source:HGNC Symbol;Acc:HGNC:20704] | 4598 | 0.105 | 0.2261 | No |
| 9 | FEM1C | fem-1 homolog C [Source:HGNC Symbol;Acc:HGNC:16933] | 4987 | 0.074 | 0.2068 | No |
| 10 | AMBRA1 | autophagy and beclin 1 regulator 1 [Source:HGNC Symbol;Acc:HGNC:25990] | 6213 | -0.022 | 0.1149 | No |
| 11 | HERPUD1 | homocysteine inducible ER protein with ubiquitin like domain 1 [Source:HGNC Symbol;Acc:HGNC:13744] | 6520 | -0.044 | 0.0975 | No |
| 12 | KLHDC10 | kelch domain containing 10 [Source:HGNC Symbol;Acc:HGNC:22194] | 6687 | -0.055 | 0.0926 | No |
| 13 | FEM1B | fem-1 homolog B [Source:HGNC Symbol;Acc:HGNC:3649] | 8065 | -0.160 | 0.0091 | No |
| 14 | APPBP2 | amyloid beta precursor protein binding protein 2 [Source:HGNC Symbol;Acc:HGNC:622] | 8134 | -0.165 | 0.0280 | No |
| 15 | SKP1 | S-phase kinase associated protein 1 [Source:HGNC Symbol;Acc:HGNC:10899] | 8454 | -0.190 | 0.0311 | No |
| 16 | BTRC | beta-transducin repeat containing E3 ubiquitin protein ligase [Source:HGNC Symbol;Acc:HGNC:1144] | 10836 | -0.409 | -0.0940 | No |
| 17 | TRPC4AP | transient receptor potential cation channel subfamily C member 4 associated protein [Source:HGNC Symbol;Acc:HGNC:16181] | 11053 | -0.438 | -0.0467 | No |
| 18 | ZSWIM8 | zinc finger SWIM-type containing 8 [Source:HGNC Symbol;Acc:HGNC:23528] | 11287 | -0.470 | 0.0038 | No |
| 19 | SPSB1 | splA/ryanodine receptor domain and SOCS box containing 1 [Source:HGNC Symbol;Acc:HGNC:30628] | 12654 | -0.830 | 0.0191 | No |
Table: GSEA details [plain text format]

  

Fig 2: GOMF\_UBIQUITIN\_LIGASE\_SUBSTRATE\_ADAPTOR\_ACTIVITY      
 Blue-Pink O' Gram in the Space of the Analyzed GeneSet

  

Fig 3: GOMF\_UBIQUITIN\_LIGASE\_SUBSTRATE\_ADAPTOR\_ACTIVITY: Random ES distribution      
 Gene set null distribution of ES for **GOMF\_UBIQUITIN\_LIGASE\_SUBSTRATE\_ADAPTOR\_ACTIVITY**
